# Emery-Dreifuss Muscular Dystrophy: a Report of a Large Family with 11 Affected Individuals

**Published:** 2016-09-04

**Authors:** Azadeh Ahmadifard, Javad Jamshidi, Abbas Tafakhori, Reza Mollazadeh, Zeinab Falsafi, Hossein Darvish

**Affiliations:** 1 *Department of Medical Genetics, School of Medicine, Shahid Beheshti University of Medical Sciences, Tehran, Iran.*; 2 *Noncommunicable Diseases Research Center, Fasa University of Medical Sciences, Fasa, Iran.*; 3 *Department of Neurology, School of Medicine, Imam Khomeini Hospital and Iranian Center of Neurological Research, Tehran University of Medical Sciences, Tehran, Iran.*; 4 *Electrophysiology and Pacemarker, Cardiology Department, Imam Khomeini Hospital, Tehran University of Medical Sciences, Tehran, Iran.*

**Keywords:** Emery-Dreifuss, muscular dystrophy, X-linked, *EMD*, Iranian

To the Editor,

Emery-Dreifuss muscular dystrophy (EDMD) is a rare condition which mainly affects the skeletal and cardiac muscles(1). Cardiac conduction defects usually occur and almost all patients have heart problems by adulthood. These cardiac abnormalities can lead to bradycardia, fainting, and an increased risk of stroke and sudden death (2). One of the earliest symptoms are contractures, which restrict the movement of certain joints. Most affected individuals also experience slowly progressive muscle weakness and wasting (3). EDMD is genetically heterogeneous and can occur in X-linked dominant (X-LD), autosomal dominant (AD) and autosomal recessive (AR) forms. The X-LD is the most common type which is usually caused by mutation in *EMD *or* FHL1* genes (4). *EMD *gene is located on Xq28 and encodes emerin, which is a serine- rich nuclear membrane protein and a member of the nuclear lamina- associated protein family (5). Here we describe a large family from Iran with EMDM which have 11 affected men (Figure 1A).

The patients were normal at birth with normal milestones. The proband (II.2) had a chronic progressive muscle weakness from his second decade of life. He had normal mental examination state and severe cardiac arrhythmia that resulted in pacemaker implantation.

Distribution of weakness was proximal and distal; but distal muscles were more atrophic and weak. He had some contractures in elbow and also had rigid spine. There was no ptosis or severe facial weakness. Deep tendon reflexes diminished. Muscle tone was normal but severe contractions interfered with examination. Humeral muscle atrophy and elbow contracture were also seen which is pathognomonic for EMDM (Figure 1B). There was nothing abnormal in ophthalmologic examination. Two of the patients in the second generation died suddenly at the 4^th ^decade of life due to cardiac problems. Echocardiography (ECG) showed mild left ventricular systolic dysfunc-tion. ECG showed functional escape rhythm at the rate of 40/min. Curiously, atrial activity in the form of obvious P wave or even atrial fibrillation was absent, which correspond to atrial standstill.

As the patients were diagnosed with EMDM, and the pedigree showed an X-linked pattern of inheritance, *EMD *gene was sequenced to detect the possible mutations. A nonsense mutation was detected in exon 4 of *EMD* gene in hemizygous form in affected men (c.315T>A, p.Y105*) (Figure 1C). Although this mutation in protein level was previously reported (6), our family had a novel nucleotide substitution mutation (TAT>TAA in codon 105 of EMD gene vs. TAT>TAG in previous

report).

The severity of X-linked EMDM caused by mutations in *EMD* gene could be different. Sometimes this difference can be attributed to the different mutations in the gene. However, the same mutations in different families have been reported with different severities (6, 7). Cardiac conduction defect is one of the most important symptoms associated with *EMD* mutations which was present in our patients. Some studies even reported EMD mutations with isolated atrial cardiac disease with conduction abnormalities (8). Our patients present the classic symptoms of EMDM, which is similar to some previously reported nonsense mutations in *EMD* gene (6, 9, 10).

**Fig. 1:**
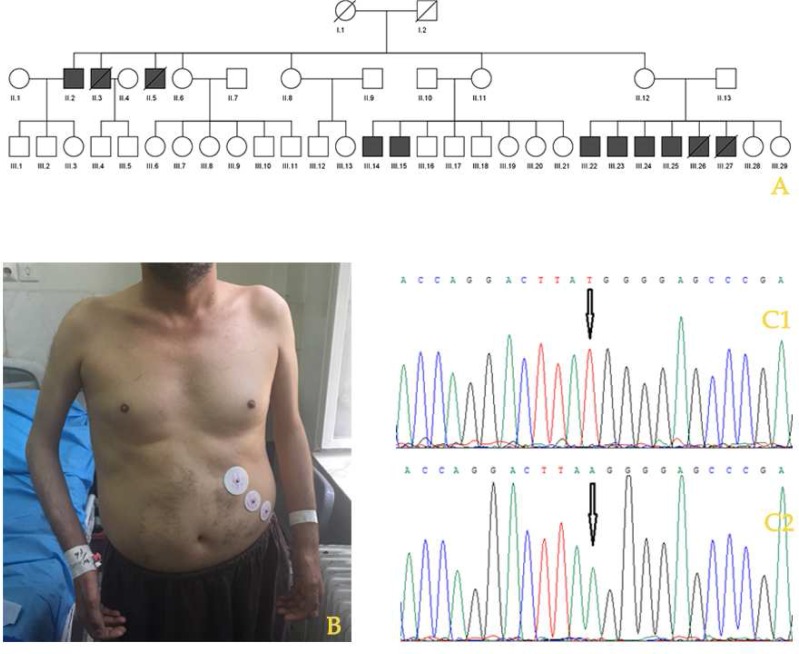
Phenotype, pedigree and mutation analysis of an EMDM patient. A: pedigree of the studied family; B: an affected male in the family who shows humeral muscle atrophy and elbow contracture; C1: normal sequence; C2: a sequence from an affected male (the arrow indicates the location of the mutation (c.315T>A)).

Here we described one of the largest EMDM families reported so far. 11 men were affected in this family with a nonsense mutation in *EMD* gene. The mutated gene was inherited from their carrier mothers. To the best of our knowledge, this is also the first report of genetic study of EMDM from Iran.

## Conflict of interest

The authors declared no conflict of interest.
